# Effect of Difference in Serum Creatinine between Jaffe and Enzymatic Methods in Outpatient Kidney Transplant Recipients

**DOI:** 10.3390/jcm13206066

**Published:** 2024-10-11

**Authors:** Kristina Boss, Susanne Stolpe, André Müller, Justa Friebus-Kardash, Bernd Wagner, Marc Wichert, Roland Assert, Lothar Volbracht, Andreas Stang, Bernd Kowall, Andreas Kribben

**Affiliations:** 1 Department of Nephrology, University Hospital Essen, University Duisburg-Essen, 45147 Essen, Germany; 2 Institute of Medical Informatics, Biometry and Epidemiology, University Hospital Essen, University Duisburg-Essen, 45147 Essen, Germany; 3 Department of Clinical Chemistry, University Hospital Essen, University Duisburg-Essen, 45147 Essen, Germany

**Keywords:** chronic kidney disease, creatinine, enzymatic method, Jaffe method, kidney transplantation

## Abstract

**Highlights:**

**What was known:**

- Deviations in serum creatinine (SCr), due to its determination using a Jaffe or an enzymatic method, have an effect on kidney disease detection and staging.
- It is not yet clear how large this effect is in kidney transplant recipients (KTRs).
- SCr measurement differences are of particular importance here to evaluate the graft function.

**This study adds:**

- Investigation of the difference between Jaffe SCr and enzymatic SCr in a large cohort of outpatient KTRs.
- In more than every tenth SCr determination in outpatient KTRs, the difference between the Jaffe and enzymatic methods had an influence on the assignment to a CKD stage.
- This effect was comparably pronounced for all eGFR formulas applied.

**Potential impact:**

- Clinicians should keep in mind that the difference between Jaffe and enzymatic SCr affects CKD staging in KTRs.

**Abstract:**

**Background:** Deviations in serum creatinine (SCr), due to its determination using a Jaffe or an enzymatic method, have an effect on kidney disease detection and staging. It is not yet clear how large this effect is in kidney transplant recipients (KTRs). SCr measurement differences are of particular importance here to evaluate the graft function. **Methods:** The results of all parallel SCr measurements (Jaffe and enzymatic method) of adult outpatient KTRs in the same serum sample at the University Hospital Essen (Germany) between January 2020 and October 2023 were evaluated. A Bland–Altman plot with 95% limits of agreement (LoA) was used to assess the difference between the Jaffe and the enzymatic SCr (eSCr). For all patients, we used the CKD-EPI 2009 and EKFC formula, and for patients ≥ 70 years, we also used the BIS1 formula for the determination of eGFR. **Results:** A total of 12,081 parallel SCr measurements from 1243 KTRs were analyzed, where 61% were male and the median age was 53 years. On average, Jaffe SCr was 0.03 mg/dL higher than eSCr (LoA −0.16; 0.21 mg/dL). On average, the eGFR determined by Jaffe SCr was 1.9 mL/min/1.73 m^2^ lower than the eGFR determined by eSCr (LoA −9.5; 5.7 mL/min/1.73 m^2^). The comparison of eGFR between the two SCr methods revealed a different CKD stage in 1589 (13%) of all analyzed measurements, most frequently between G2/G3a (41%) and G3a/G3b (24%). When using the EKFC and BIS1 formulas, there were approximately the same number of measurements leading to a different CKD stage. **Conclusions:** In more than every tenth SCr determination in outpatient KTRs, the difference between the Jaffe and enzymatic methods had an influence on the assignment to a CKD stage. This effect was comparably pronounced for all eGFR formulas applied.

## 1. Introduction

Kidney transplantation is a kidney replacement therapy that is associated with lower morbidity and mortality than chronic dialysis [1]. Two decisive factors for a long graft survival are delaying of chronic kidney disease (CKD) progression and early detection of rejections. To delay CKD progression and its associated complications, patients are treated with a comprehensive treatment strategy. This includes the use of progression-delaying drugs like sodium-glucose cotransporter-2 inhibitors (SGLT2is) and avoidance or dose adaption of nephrotoxic drugs like nonsteroidal anti-inflammatory drugs (NSAIDs), vancomycin, or proton pump inhibitors [2,3].

Determination of estimated glomerular filtration rate (eGFR) is the essential method to define the current CKD stage and to monitor kidney (graft) function and thus the basis for decisions on therapies. The same applies to the detection of rejections. Slight changes in graft function are often the first sign of a rejection. The most accurate estimation of the GFR depends on two factors: the most precise measurement of serum creatinine (SCr) and the most precise formula for estimating the GFR.

There has been some progress in this area in recent years. Standardized alkaline picrate (Jaffe) assays and enzymatic assays are mainly used to measure SCr in automated platforms in clinical laboratories [4]. In addition to the CKD-EPI 2009 eGFR formula, the European Kidney Function Consortium (EKFC) eGFR formula has also become increasingly important [5]. In the current KDIGO guideline, the EKFC formula is recommended as equal to the CKD-EPI formula for the determination of the kidney function [4]. The advantages of the EKFC formula lie in its applicability to patients between 2 and 100 years of age, so that there is no physiologically implausible change in eGFR because a different formula is used from the age of 18. In addition, the EKFC formula was developed in study populations of predominantly European subjects, which is more reflective of patients treated in Europe. Also, the EKFC formula is based on a different mathematical modeling: by using a percentile distribution of SCr and/or cystatin c in the healthy population, the influencing factors of age and gender are already adjusted at the biomarker level and not, as in the CKD-EPI formula, only at the GFR level [6].

Despite this progress, there are still SCr differences due to measurement by the Jaffe or enzymatic method. These differences have an effect on kidney disease detection and staging. In 19% of all cases, there was a different CKD stage when comparing eGFRs between the two SCr methods, of which 98% resulted in a more severe CKD stage determined with Jaffe SCr [7]. In kidney transplant recipients (KTRs), it is particularly important to determine SCr and the eGFR as accurately as possible to assess graft function and detect a rejection. Even a small increase in SCr or a gradual decrease in eGFR can be decisive for the transplant physician, e.g., to initiate further diagnostics or to shorten the intervals between check-ups. It is not yet clear how large the effect of different SCr measurement methods and the use of different eGFR formulas is in KTRs, i.e., to what extent this problem also applies to KTRs. The aim of this study was therefore to investigate the difference between Jaffe SCr and eSCr in a large cohort of outpatient KTRs and to evaluate to what extent a possible difference between the results of the measurement methods affects the staging of CKD.

## 2. Material and Methods

### 2.1. Study Design

We analyzed all serum creatinine measurements of outpatient adult KTRs of the Department of Nephrology at the University Hospital Essen, Essen, Germany between January 2020 and October 2023, in which creatinine was determined using both the Jaffe and enzymatic methods.

The two SCr measurements were carried out simultaneously. The University Hospital Essen in Essen, Germany’s Department of Clinical Chemistry conducted analyses on all SCr measurements. Atellica measurement systems (Atellica 930 analyser, Atellica CH Crea_2 assay, Siemens Healthcare Diagnostics, Marburg, Germany) were used to correlate patient samples and reference material SRM967 from the National Institute of Standards and Technology (NIST) in order to determine Jaffe SCr using the same IDMS traceable method over the course of this study. The Atellica measurement systems and the same IDMS traceable method were used to determine the eSCr. NIST SRM967 can also be traced back to the assay. The laboratory procedure thus corresponded to that of the previous study [7].

The current KDIGO guidelines [4] were used to define CKD. The eGFR was calculated using the CKD-EPI 2009 formula for SCr and was used as a measure of renal function [8]. Furthermore, the BIS1 formula was utilized for patients over 70 years of age, and the EKFC eGFR formula was applied to all patients [5,9].

### 2.2. Statistical Analysis

The study population was described through the analysis of frequency distributions, central tendency, and variability measurements. The SCr difference between the two measurement techniques (SCr determination by Jaffe and enzymatic methods) was calculated using a Bland–Altman plot. As a sensitivity analysis, we analyzed only the first parallel SCr (Jaffe and eSCr) measurement per patient. The agreement between the stages of CKD classification was assessed using kappa values [10]. SAS (version 9.4; Cary, NC, USA) and GraphPad Prism (version 10.2.2; San Diego, CA, USA) software were used for all statistical analyses and graphical evaluations.

### 2.3. Ethical Approval

The International Conference on Harmonization’s Good Clinical Practice guidelines and the Declaration of Helsinki were followed during this study’s execution. The local ethics committee of the University of Duisburg-Essen approved this study (20-9501-BO, ethical approval date 12 August 2020).

## 3. Results

### 3.1. Study Population Characteristics

A total of 12,081 parallel SCr measurements of 1243 outpatient kidney transplant recipients were evaluated. Male patients made up 61% of the cohort. The age range was 18–90 years, with a median age of 53. The mean age was 50 years. The median frequency was six measurements per patient (IQR 3–14; range 1–74; Supplement Figure S1).

### 3.2. Impact of the Measurement Method on Serum Creatinine

**Figure 1:**
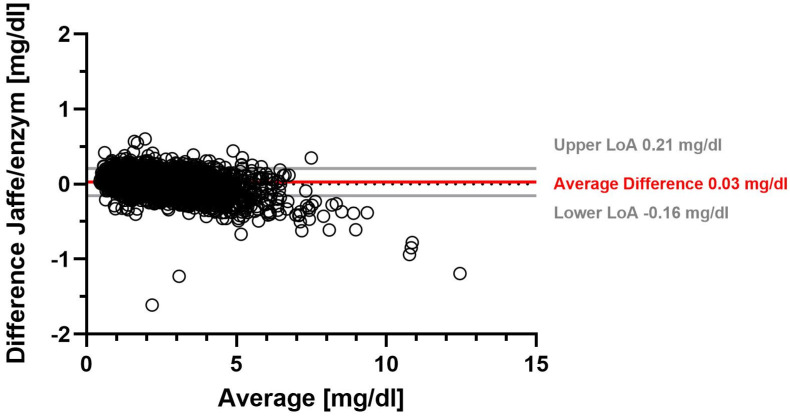
Serum creatinine difference between Jaffe and enzymatic measurement methods.

Using the Jaffe method, the overall mean SCr was 1.78 mg/dL with a median SCr of 1.52 mg/dL. The overall mean enzymatic SCr was 1.76 mg/dL (median 1.48 mg/dL) (Supplementary Table S1). On average, SCr determined with the Jaffe method was 0.03 mg/dL higher than SCr determined with the enzymatic method. Table 1 shows that the range of −0.16 mg/dL to 0.21 mg/dL accounted for 95% of the differences in SCr between the Jaffe and enzymatic methods. A Bland–Altman plot illustrates the difference between the two SCr measurement methods for all measurements (Figure 1). We found the largest average difference between Jaffe and eSCr in females, with 0.04 mg/dL (Table 1, Supplementary Table S3).

Figure 1 shows a Bland–Altman plot of the difference between serum creatinine determined by the Jaffe method and by the enzymatic method. Solid gray lines represent upper and lower limits of agreement (LoA), and the solid red line represents the average difference, while the dotted gray line marks the zero line.

### 3.3. Impact of the Measurement Method on the Estimated Glomerular Filtration Rate

**Figure 2:**
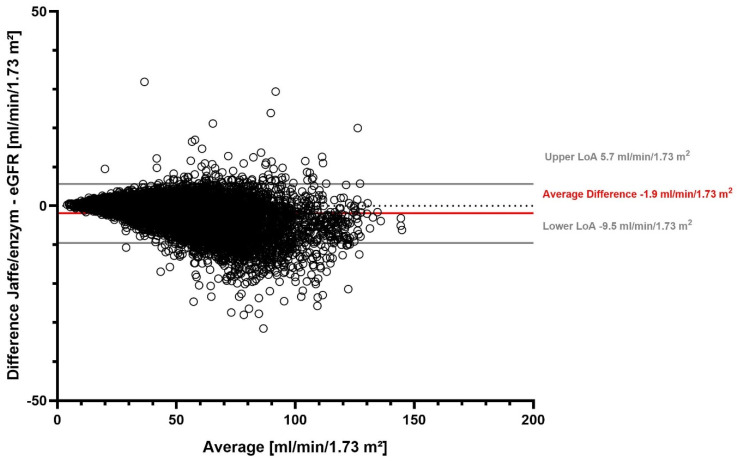
eGFR difference between Jaffe and enzymatic serum creatinine measurement methods.

The overall mean eGFR with the Jaffe method was 48.9 mL/min/1.73 m^2^ (median 47.3 mL/min/1.73 m^2^), and it was 50.8 mL/min/1.73 m^2^ with the enzymatic method (median 49.0 mL/min/1.73 m^2^) (Supplementary Table S2). On average, the eGFR difference was −1.9 mL/min/1.73 m^2^. We found that 95% of the differences in eGFR between the two SCr measurement methods fell in the range between −9.5 mL/min/1.73 m^2^ and 5.7 mL/min/1.73 m^2^ (Table 2). Our stratified analyses showed that the largest average difference was detected in females, while the lowest average difference in patients ≥ 80 years old (Table 2, Supplementary Table S3). The eGFR difference between the two SCr measurement methods for all measurements is visualized in a Bland–Altman plot (Figure 2).

Figure 2 shows a Bland–Altman plot of the difference between eGFR determined with the Jaffe and enzymatic serum creatinine methods. Solid gray lines represent the upper and lower limits of agreement (LoA), the solid red line represents the average difference, and the dotted gray line marks the zero line.

### 3.4. Impact of the Measurement Method on CKD Staging

Patients in all CKD stages were included in this study (Supplementary Table S4a,b). The SCr difference, and thus the difference in eGFR, due to the two different creatinine measurement methods had an impact on the staging of CKD. When utilizing Jaffe or enzymatic SCr for determining eGFR with the CKD-EPI formula, there were upgrading (less severe CKD stage) and downgrading (more severe CKD stage) effects with a switch of CKD stage in 13% (n = 1589) of cases. Among these 1589 disagreements of CKD stage, 99.7% were disagreements between adjacent CKD stages. The largest number of different CKD classifications was between G2/G3a (41%) and G3a/G3b (24%) (Table 3, Table 4 and Table 5). We found a kappa value of 0.83 (95% CI 0.83–0.84).

### 3.5. Impact of the Measurement Method on Adults ≥ 70 Years

For all patients ≥ 70 years old, the CKD-EPI and the BIS1 eGFR formula were applied with Jaffe and eSCr. In 9.9% of the measurements, a deviating CKD classification was obtained using the CKD-EPI eGFR formula with Jaffe and eSCr. When the BIS1 formula was used, there was approximately the same proportion of variation in the resulting CKD stages (9.1%). The kappa values here were also similar, at 0.86 (95% CI 0.84–0.89) and 0.86 (95% CI 0.83–0.88), respectively (Supplementary Tables S5a,b and S6). Nearly one half of the measurements with CKD stage G2 according to eSCr had CKD stage G3a according to the corresponding Jaffe SCr, but the number of measurements was very small.

### 3.6. Impact of the Measurement Method on CKD Stages When Applying the EKFC Formula

Using Jaffe und eSCr in conjunction with the EKFC eGFR formula, 1488 (12.3%) of the measurements yielded a deviating CKD classification, which was approximately the same proportion of deviations as when applying the CKD-EPI eGFR formula (Table 6 and Table 7). The Kappa value here was 0.84 (95% CI 0.83–0.85) and thus approximately in the same range as when applying the CKD-EPI formula.

## 4. Discussion

### 4.1. Key Findings

To our knowledge, this is the first study to have investigated the effect of a difference between Jaffe SCr and eSCr in a large cohort of outpatient KTRs. We found that the absolute average SCr difference was small. But the resulting differences in eGFR led to clinically highly relevant effects regarding CKD classification: in more than every tenth SCr determination in outpatient KTRs, the difference between the Jaffe and enzymatic methods resulted in different CKD stages, most frequently between G2/G3a (41%) and G3a/G3b (24%). When using the EKFC and BIS1 formulas, there were approximately the same numbers of cases with different CKD stages.

### 4.2. Comparison with Previous Studies and Prospects

Several studies have evaluated the effect of the SCr measurement method on the determination of eGFR and the staging of CKD in different subpopulations [11,12,13,14]. The authors have come to diverse conclusions as to how clinically relevant the observed measurement differences are. A major limitation of these studies is the small number of patients investigated. We ourselves demonstrated in a comprehensive cohort study that differences between Jaffe SCr and eSCr may very well have clinical relevance in relation to detection and staging of kidney diseases [7]. In this study, we found the largest proportion of different CKD classifications between stages G1/G2 and G3a/G3b. Deviating eGFR results corresponding to CKD stage G3a or higher had the greatest clinical impact: more than every fifth patient with CKD G3a according to eSCr had CKD G3b or higher according to the corresponding Jaffe SCr [7]. In the study here, we investigated KTRs as a subgroup in which a precise determination of kidney function is of particular relevance. The average difference between Jaffe SCr and eSCr was lower in outpatient KTRs than in the cohort of all outpatients of the university hospital (0.03 mg/dL vs. 0.07 mg/dL). SCr differences between the Jaffe and enzymatic methods also resulted in different CKD stages, most frequently between G2/G3a and G3a/G3b and thus with higher-grade CKD stages than in the total cohort.

In recent years, various new eGFR formulas have been developed to estimate the glomerular filtration rate even more precisely. These include the BIS and the EKFC formulas. In a mixed patient cohort, the number of cases with a CKD switch in stages G3a/G3b in patients >70 years of age was substantially lower when applying the BIS1 eGFR equation [7]. On the other hand, the number of cases with a switch between these stages was higher when applying the EKFC eGFR equation when compared with applying the CKD-EPI equation [7]. We did not observe these effects in outpatient KTRs. When using the EKFC and BIS1 formulas, there were approximately the same numbers of cases with different CKD stages and a higher level of agreement based on the kappa values.

Our findings underline that there is no “one eGFR formula fits all” approach in everyday clinical practice. The respective advantages and disadvantages of an eGFR formula must be known and weighed up for each individual patient so that the most precise formula can finally be used. This was recently made impressively clear in an editorial by Paul Williams, who spoke out clearly in favor of stratified analyses and patient-specific selection of the best model [15].

A step beyond demographic-based eGFR formulas is the approach of Stehlé et al., who developed a formula based on serum creatinine and muscle mass assessed by a CT scan [16]. This MMB-eGFR displayed better performance levels than CKD-EPI and the EKFC eGFR formula, especially in patients with chronic diseases or discordant creatinine and cystatin C-based eGFR, thus presumed to have atypical non-GFR determinants of creatinine. Both characteristics apply to KTRs, so this could be an interesting new approach for the future.

### 4.3. Strengths and Limitations

Our study has several strengths and limitations. To our knowledge, this is the first study that has evaluated the effect of the serum creatinine difference between the Jaffe and the enzymatic methods in outpatient kidney transplant recipients. It showed a clinically relevant effect regarding CKD staging, with a switch in CKD stage in more than every tenth SCr measurement. For most patients, we included more than one measurement per patient in the evaluation. The sensitivity analysis (including only the first parallel SCr measurement per patient) showed similar results (Supplementary Tables S7–S9).

Nevertheless, some limitations must be taken into account. There were no data on cystatin C, so corresponding eGFR formulas could not be applied. Also, we used Jaffe SCr to apply the CKD-EPI, EKFC, and BIS eGFR equations. These eGFR equations have been validated only using eSCr, but it is very likely when determining Jaffe SCr in a clinical context that this measurement result is used for estimating GFR. Another limitation is the lack of measured GFR with the gold-standard technique. In the six CKD stages, a difference of varying size between the measurement methods is possible. This is shown by the shape of the Bland–Altman plot, which should have a funnel shape. However, there are significantly fewer measured values in CKD stage G1 than in the higher CKD stages, so the funnel shape is not recognizable in all parts of the plot.

Furthermore, we did not check for HIL indices in this study. Both Jaffe SCr and eSCr could possibly increase due to interferences, e.g., by glucose, bilirubin, or drugs. The serum levels of these parameters were not assessed in this study. Analyses of HIL indices were performed in our previous study and showed extremely high concentrations of, e.g., conjugated bilirubin in less than 1% of the measurements [7]. Nevertheless, it is possible that, e.g., diabetic patients with high serum glucose levels show a different degree of difference between Jaffe and enzymatic serum creatinine than non-diabetic patients.

## 5. Conclusions

In about one in seven SCr determinations in outpatient kidney transplant recipients, the difference between the Jaffe and enzymatic methods had an impact on the assignment to a CKD stage. The proportions of different CKD stages were about the same between the CKD-EPI, EKFC, and BIS1 eGFR formulas.

## Figures and Tables

**Table 1:** Average difference (Jaffe/enzymatic SCr) in serum creatinine stratified by sex and age of outpatient kidney transplant recipients at the University Hospital Essen, Essen, Germany.

	N	Average Difference SCr	Upper Limit SCr	Lower Limit SCr
**all**	12,081	0.03	0.21	−0.16
male	7370	0.02	0.22	−0.18
female	4711	0.04	0.19	−0.12
**age**				
18–29	1434	0.00	0.21	−0.20
30–39	2159	0.02	0.18	−0.13
40–49	1545	0.03	0.21	−0.15
50–59	2888	0.03	0.21	−0.15
60–69	2809	0.03	0.22	−0.15
70–79	1134	0.02	0.22	−0.18
≥80	112	0.01	0.17	−0.14

Abbreviations: SCr, serum creatinine. Average difference (Jaffe—enzymatic Sc), upper and lower limits of agreement (LoA) are presented in mg/dL. Strati and “N” reflect the number of measurements.

**Table 2:** Average difference (Jaffe/enzymatic) in estimated glomerular filtration rate stratified by sex and age of outpatient kidney transplant recipients at the University Hospital Essen, Essen, Germany.

	N	Average Difference eGFR	Upper Limit eGFR	Lower Limit eGFR
**All**	12,081	−1.9	5.7	−9.5
Male	7370	−1.6	5.5	−8.6
Female	4711	−2.5	5.7	−10.8
**Age**				
18–29	1434	−1.5	6.4	−9.4
30–39	2159	−2.1	6.2	−10.3
40–49	1545	−2.4	5.7	−10.6
50–59	2888	−2.3	5.5	−10.0
60–69	2809	−1.9	5.2	−8.9
70–79	1134	−1.1	4.6	−6.7
≥80	112	−0.7	3.2	−4.7

Abbreviations: eGFR estimated glomerular filtration rate. Average difference (Jaffe/enzymatic SCr), upper and lower limits of agreement (LoA) are presented in ml/min/1.73 m^2^. eGFR is according to the CKD-EPI formula 2009 [8]. Strati and “N” reflect the number of measurements.

**Table 3:** Average difference in serum creatinine level and estimated glomerular filtration rate stratified by CKD stages of adult outpatient kidney transplant recipients at the University Hospital Essen, Essen, Germany.

	N	Average Difference eGFR	Upper Limit eGFR	Lower Limit eGFR	Average Difference SCr	Upper Limit SCr	Lower Limit SCr
**CKD stage**							
G1	761	−1.4	28.3	−31.1	0.02	0.43	−0.39
G2	3218	−1.0	20.4	−22.3	0.02	0.48	−0.45
G3a	2811	−0.2	11.5	−11.8	0.01	0.58	−0.57
G3b	2664	−0.1	11.9	−12.0	0.01	0.88	−0.87
G4	2235	0.0	11.6	−11.6	0.00	1.62	−1.63
G5	392	0.1	6.4	−6.3	0.00	3.30	−3.40

Abbreviations: SCr, serum creatinine; eGFR, estimated glomerular filtration rate. Average difference (Jaffe/enzymatic SCr/eGFR), upper and lower limits of agreement (LoA) are presented in mg/dL and ml/min/1.73 m^2^, respectively. eGFR is according to the CKD-EPI formula 2009 [8]. Assignment to CKD stage is according to enzymatic SCr. Strati and “N” reflect the number of measurements.

**Table 4:** CKD stages depending on SCr measurement method (CKD-EPI eGFR formula).

	ENZYM	G1	G2	G3A	G3B	G4	G5	TOTAL
JAFFE								
G1		512	21	0	0	0	0	533
G2		249	2654	104	0	0	0	3007
G3A		0	543	2389	68	1	0	3001
G3B		0	0	318	2467	101	0	2886
G4		0	0	0	126	2116	38	2280
G5		0	0	0	3	17	354	374
TOTAL		761	3218	2811	2664	2235	392	12,081

Abbreviations: SCr, serum creatinine; eGFR, estimated glomerular filtration rate. eGFR is according to the CKD-EPI formula 2009 [8]. We collected 1589 measurements with different CKD stages due to the difference between Jaffe and enzymatic SCr, e.g., for G2/G3a: (543 + 104)/1589 = 40.7%.

**Table 5:** Proportion of measurements with a higher CKD stage due switching from enzymatic SCr to Jaffe SCr (CKD-EPI eGFR formula).

G1	G2+	32.7%
G2	G3a+	16.9%
G3a	G3b+	11.3%
G3b	G4+	4.8%
G4	G5	0.8%

Abbreviations: underlying estimated glomerular filtration rate (eGFR) is according to the CKD-EPI formula 2009 [8]. In orange background, CKD stage with enzymatic serum creatinine; in gray background, switched CKD stages with Jaffe serum creatinine. Percentage numbers show proportion of deviated CKD classification (e.g., 32.7% of all measurements with CKD stage G1 according to enzymatic SCr had CKD stage G2 or even higher CKD stages according to the corresponding Jaffe SCr -> 249/761 = 32.7%).

**Table 6:** CKD stages depending on SCr measurement method (EKFC eGFR formula).

	ENZYM	G1	G2	G3A	G3B	G4	G5	TOTAL
JAFFE								
G1		379	23	0	0	0	0	402
G2		188	2705	76	0	0	0	2969
G3A		0	519	2495	75	1	0	3090
G3B		0	0	331	2529	94	0	2954
G4		0	0	0	143	2173	25	2341
G5		0	0	0	0	13	312	325
TOTAL		567	3247	2902	2747	2281	337	**12,081**

Abbreviations: SCr, serum creatinine; eGFR, estimated glomerular filtration rate. eGFR is according to the EKFC formula [8].

**Table 7:** Proportion of measurements with a higher CKD stage due to the use of Jaffe SCr (EKFC eGFR formula).

G1	G2+	33.2%
G2	G3a+	16.0%
G3a	G3b+	11.4%
G3b	G4+	5.2%
G4	G5	0.6%

Abbreviations: Underlying estimated glomerular filtration rate (eGFR) according to the EKFC formula [5]. In orange background, CKD stage with enzymatic serum creatinine; in gray background, switched CKD stages with Jaffe serum creatinine. Percentage numbers show proportion of deviated CKD classification (e.g., 33.2% of all measurements with CKD stage G1 according to enzymatic SCr had CKD stage G2 or even higher CKD stages according to the corresponding Jaffe SCr).

## Data Availability

The datasets presented in this article are not readily available because the data are part of an ongoing study. Requests to access the datasets should be directed to the corresponding author.

## Supplementary Materials

The following supporting information can be downloaded at: https://www.mdpi.com/article/10.3390/jcm13206066/s1, Figure S1: Frequency distribution of serum creatinine measurements per patient; Table S1: Serum creatinine stratified by sex and age; Table S2: Estimated glomerular filtration rate stratified by sex and age; Table S3: Average difference in serum creatinine and estimated glomerular filtration rate stratified by sex and age; Table S4a: Serum creatinine stratified by CKD stages according to enzymatic SCr; Table S4b: Estimated glomerular filtration rate stratified by CKD stages according to enzymatic SCr; Table S5a: CKD stages depending on SCr measurement method when applying the CKD-EPI eGFR formula in patients ≥ 70 years; Table S5b: CKD stages depending on SCr measurement method when applying the BIS1 eGFR formula in patients ≥ 70 years; Table S6: Deviation of CKD stages depending on eGFR formula in patients > 70 years; Table S7: Average difference (Jaffe/enzymatic SCr) of serum creatinine stratified by sex (only first parallel SCr measurement); Table S8: Serum creatinine stratified by sex (only first parallel SCr measurement); Table S9: CKD stages depending on SCr measurement method when applying the CKD-EPI eGFR formula (only first parallel SCr measurement).

## Abbreviations

CKD	chronic kidney disease
eGFR	estimated glomerular filtration rate
eSCr	enzymatic serum creatinine
EKFC	European Kidney Function Consortium
HIL	hemolysis, icterus, and lipemia
KTR	kidney transplant recipient
NSAIDs	nonsteroidal anti-inflammatory drugs
SCr	serum creatinine
SGLT2is	sodium-glucose cotransporter-2 inhibitors
