# Supplementary material for: Effect of Difference in Serum Creatinine between Jaffe and Enzymatic Methods in Outpatient Kidney Transplant Recipients
Source: J Clin Med. 2024 Oct 11;13(20):6066. doi: 10.3390/jcm13206066 (PMC11508460; doi:10.3390/jcm13206066)
Supplement: Supplementary file 1 [file jcm-13-06066-s001.zip › jcm-3232469-supplementary.pdf]

Supplement

Figure S1	Frequency distribution of serum creatinine measurements per patient
Table S1	Serum creatinine stratified by sex and age
Table S2	Estimated glomerular filtration rate stratified by sex and age
Table S3	Average difference in serum creatinine and estimated glomerular filtration rate stratified by sex and age
Table S4a	Serum creatinine stratified by CKD stages according to enzymatic SCr
Table S4b	Estimated glomerular filtration rate stratified by CKD stages according to enzymatic SCr
Table S5a	CKD stages depending on SCr measurement method when applying the CKD-EPI eGFR formula in patients ≥ 70 years
Table S5b	CKD stages depending on SCr measurement method when applying the BIS1 eGFR formula in patients ≥ 70 years
Table S6	Deviation of CKD stages depending on eGFR formula in patients > 70 years
Table S7	Average difference (Jaffe / enzymatic SCr) of serum creatinine stratified by sex (only first parallel SCr measurement)
Table S8	Serum creatinine stratified by sex (only first parallel SCr measurement)
Table S9	CKD stages depending on SCr measurement method when applying the CKD-EPI eGFR formula (only first parallel SCr measurement)

Supplementary Figure S1: Frequency distribution of serum creatinine measurements per patient.

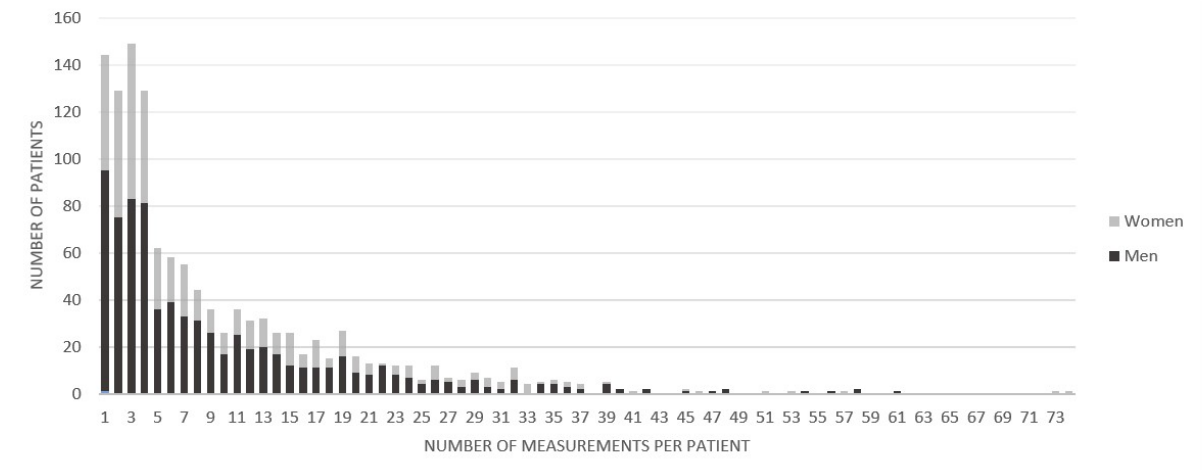

Supplementary Table S1: Serum creatinine stratified by sex and age.

	N	Mean SCr Jaffe / enzymatic	Median SCr Jaffe / enzymatic	SD SCr Jaffe / enzymatic
**all**	12,081	1.78 / 1.76	1.52 / 1.48	0.9 / 0.9
male	7,370	1.97 / 1.95	1.67 / 1.64	0.9 / 1.0
female	4,711	1.49 / 1.46	1.31 / 1.26	0.7 / 0.7

**age**				
18-29	1,434	1.92 / 1.91	1.52 / 1.50	1.1 / 1.2
30-39	2,159	1.56 / 1.53	1.39 / 1.36	0.8 / 0.8
40-49	1,545	1.77 / 1.74	1.47 / 1.43	0.9 / 0.9
50-59	2,888	1.78 / 1.74	1.49 / 1.44	0.9 / 0.9
60-69	2,809	1.81 / 1.78	1.58 / 1.54	0.9 / 0.9
70-79	1,134	1.99 / 1.97	1.78 / 1.76	0.9 / 0.9
≥ 80	112	1.92 / 1.91	1.80 / 1.77	0.9 / 0.9

Abbreviations: SCr, serum creatinine. Mean and median are presented in mg/dl.
Some of the data contain several measurements per patient. Strati and "N" reflect the number of measurements.

Supplementary Table S2: Estimated glomerular filtration rate stratified by sex and age.

	N	Mean eGFR Jaffe / enzymatic	Median eGFR Jaffe / enzymatic	SD eGFR Jaffe / enzymatic
**all**	12,081	48.9 / 50.8	47.3 / 49.0	22.1 / 23.8
male	7,370	48.3 / 49.9	46.6 / 48.0	22.2 / 23.7
female	4,711	49.3 / 52.4	48.3 / 50.3	21.9 / 23.8
**age**				
18-29	1,434	57.8 / 59.3	55.7 / 57.1	26.7 / 28.2
30-39	2,159	59.3 / 61.3	57.5 / 59.2	22.3 / 24.1
40-49	1,545	52.0 / 54.5	51.9 / 54.3	22.3 / 24.2
50-59	2,888	47.4 / 49.7	47.1 / 49.3	19.6 / 21.6
60-69	2,809	42.1 / 44.0	40.8 / 42.0	17.6 / 19.3
70-79	1,134	35.3 / 36.4	32.7 / 33.2	16.1 / 17.4
≥ 80	112	36.3 / 37.0	30.8 / 31.5	18.3 / 19.1

Abbreviations: eGFR, estimated glomerular filtration rate. Mean and median are presented in ml/min/1.73 m^2. eGFR is according to the CKD-EPI formula 2009 [8]. Some of the data contain several measurements per patient. Strati and "N" reflect the number of measurements.

Supplementary Table S3: Average difference in serum creatinine and estimated glomerular filtration rate stratified by sex and age of outpatient kidney transplant recipients at the University Hospital Essen, Essen, Germany.

	Male				Female			
**Age**	**N**	**Average Difference SCr**	**Upper Limit SCr**	**Lower Limit SCr**	**N**	**Average Difference SCr**	**Upper Limit SCr**	**Lower Limit SCr**
18-29	949	0.00	0.22	-0.23	485	0.02	0.17	-0.12
30-39	1,135	0.03	0.20	-0.14	1,024	0.02	0.16	-0.12
40-49	985	0.02	0.22	-0.17	560	0.05	0.20	-0.10

50-59	1,903	0.02	0.21	-0.17	985	0.05	0.21	-0.11
60-69	1,639	0.03	0.23	-1.8	1,170	0.04	0.18	-0.11
70-79	682	0.01	0.22	-0.20	452	0.04	0.20	-0.12
≥ 80	77	0.00	0.16	-0.16	35	0.04	0.17	-0.08

Age	N	Average Difference eGFR	Upper Limit eGFR	Lower Limit eGFR	N	Average Difference eGFR	Upper Limit eGFR	Lower Limit eGFR
18-29	949	-1.2	6.3	-8.6	485	-2.1	6.6	-10.9
30-39	1,135	-2.0	5.7	-9.6	1,024	-2.2	6.7	-11.1
40-49	985	-1.8	5.5	-9.1	560	-3.5	5.6	-12.6
50-59	1,903	-1.8	5.4	-8.9	985	-3.3	5.2	-11.8
60-69	1,639	-1.6	5.3	-8.5	1,170	-2.3	5.0	-9.5
70-79	682	-0.6	4.3	-5.6	452	-1.8	4.6	-8.1
≥ 80	77	-0.7	3.8	-5.2	35	-0.8	1.6	-3.3

Abbreviations: SCr, serum creatinine. Average difference (Jaffe / enzymatic Sc), upper and lower limits of agreement (LoA) are presented in mg/dl. eGFR, estimated glomerular filtration rate. Average difference, upper and lower limits of agreement (LoA) are presented in ml/min/1.73 m^2. eGFR is according to the CKD-EPI formula 2009 [8]. Strati and "N" reflect the number of measurements.

Supplementary Table S4: Measurements of serum creatinine (4a) and estimated glomerular filtration rate (4b) stratified by CKD stages according to enzymatic SCr.

Supplementary Table S4a

CKD stages	N Jaffe / enzymatic	Mean SCr Jaffe / enzymatic	Median SCr Jaffe / enzymatic	SD SCr Jaffe / enzymatic
G1	533 / 761	0.86 / 0.83	0.85 / 0.82	0.1 / 0.2
G2	3,007 / 3,218	1.13 / 1.11	1.13 / 1.11	0.2 / 0.2
G3a	3,001 / 2,811	1.42 / 1.41	1.41 / 1.41	0.2 / 0.2
G3b	2,886 / 2,664	1.83 / 1.83	1.79 / 1.79	0.3 / 0.3
G4	2,280 / 2,235	2.76 / 2.77	2.67 / 2.68	0.6 / 0.6
G5	374 / 392	4.85 / 4.91	4.58 / 4.64	1.2 / 1.3

Abbreviations: SCr, serum creatinine. Mean and median are presented in mg/dl. Strati and "N" reflect the number of measurements.

Supplementary Table S4b

CKD stages	N Jaffe / enzymatic	Mean eGFR Jaffe / enzymatic	Median eGFR Jaffe / enzymatic	SD eGFR Jaffe / enzymatic
G1	533 / 761	102.9 / 103.5	99.9 / 100.2	10.5 / 11.1

G2	3,007 / 3,218	71.4 / 72.3	70.2 / 71.6	7.9 / 8.1
G3a	3,001 / 2,811	52.4 / 52.5	52.4 / 52.6	4.2 / 4.3
G3b	2,886 / 2,664	37.3 / 37.4	37.4 / 37.5	4.4 / 4.3
G4	2,280 / 2,235	23.3 / 23.3	23.5 / 23.6	4.2 / 4.3
G5	374 / 392	11.9 / 11.9	12.4 / 12.4	2.2 / 2.2

Abbreviations: eGFR, estimated glomerular filtration rate. Mean and median are presented in ml/min/1.73 m^2. eGFR is according to the CKD-EPI formula 2009 [8]. Strati and "N" reflect the number of measurements.

Supplementary Table S5: CKD stages depending on SCr measurement method when applying the CKD-EPI eGFR formula (5a) and BIS1 eGFR formula (5b); all measurements in patients ≥ 70 years.

Supplementary Table S5a

JAFFE \ ENZYM	G1	G2	G3A	G3B	G4	G5	TOTAL
**G1**	6	0	0	0	0	0	6
**G2**	4	122	5	0	0	0	131
**G3A**	0	18	113	4	0	0	135
**G3B**	0	0	29	409	19	0	457
**G4**	0	0	0	31	405	4	440
**G5**	0	0	0	0	9	68	77
**TOTAL**	10	140	147	444	433	72	**1,246**

Supplementary Table S5b

JAFFE \ ENZYM	G1	G2	G3A	G3B	G4	G5	TOTAL
**G1**	3	0	0	0	0	0	3
**G2**	6	39	3	0	0	0	48
**G3A**	0	33	168	5	0	0	206
**G3B**	0	0	29	540	16	0	585
**G4**	0	0	0	24	370	0	394
**G5**	0	0	0	0	1	9	10
**TOTAL**	9	72	200	569	387	9	**1246**

Supplementary Table S6: Deviation of CKD stages depending on eGFR formula in measurements in patients > 70 years.

	CKD-EPI	BIS1
G1 / G2	40.0 %	33.3 %
G2 / G3a	12.9 %	45.8 %
G3a / G3b	19.7 %	14.5 %
G3b / G4	7.0 %	4.2 %

G4 / G5	2.1 %	0.3 %
G5 / G4	5.6 %	0 %

Abbreviations: eGFR, estimated glomerular filtration rate. eGFR is according to the CKD-EPI formula 2009 [8] or BIS1 formula [9]. In orange background, CKD stage with enzymatic serum creatinine; in gray background, corresponding CKD stage with Jaffe serum creatinine. Percentage numbers show proportion of deviated CKD classification (e.g., 40.0% of all measurements with CKD stage G1 according to enzymatic SCr had CKD stage G2 according to the corresponding Jaffe SCr). Percentage numbers of upgrading effects are not shown.

Supplementary Table S7: Average difference (Jaffe / enzymatic SCr) in serum creatinine stratified by sex (only first parallel SCr measurement).

	N	Average Difference SCr	Upper Limit SCr	Lower Limit SCr
**all**	1,243	0.06	0.22	-0.11
male	766	0.05	0.22	-0.12
female	477	0.06	0.22	-0.10

Abbreviations: SCr, serum creatinine. Average difference (Jaffe / enzymatic Sc), upper and lower limits of agreement (LoA) are presented in mg/dl.

Supplementary Table S8: Serum creatinine stratified by sex (only first parallel SCr measurement).

	N	Mean SCr Jaffe / enzymatic	Median SCr Jaffe / enzymatic	SD SCr Jaffe / enzymatic
**all**	1,243	1.72 / 1.66	1.48 / 1.43	0.9 / 0.9
male	766	1.85 / 1.80	1.57 / 1.53	0.9 / 0.9
female	477	1.51 / 1.45	1.29 / 1.23	0.8 / 0.8

Abbreviations: SCr, serum creatinine. Mean and median are presented in mg/dl.

Supplementary Table S9: CKD stages depending on SCr measurement method when applying the CKD-EPI eGFR formula (only first parallel SCr measurement).

ENZYM / JAFFE	G1	G2	G3A	G3B	G4	G5	TOTAL
**G1**	73	1	0	0	0	0	74
**G2**	44	267	5	0	0	0	316
**G3A**	0	73	238	2	0	0	313
**G3B**	0	0	44	240	5	0	289
**G4**	0	0	0	23	197	3	223
**G5**	0	0	0	0	1	27	28
**TOTAL**	117	341	287	265	203	30	**1,243**

Kappa value 0.79 (95% CI 0.77–0.82).
